# Supplementary figures and images for: Thermostabilization and purification of the human dopamine transporter (hDAT) in an inhibitor and allosteric ligand bound conformation
Source: PLoS One. 2018 Jul 2;13(7):e0200085. doi: 10.1371/journal.pone.0200085 (PMC6028122; doi:10.1371/journal.pone.0200085)

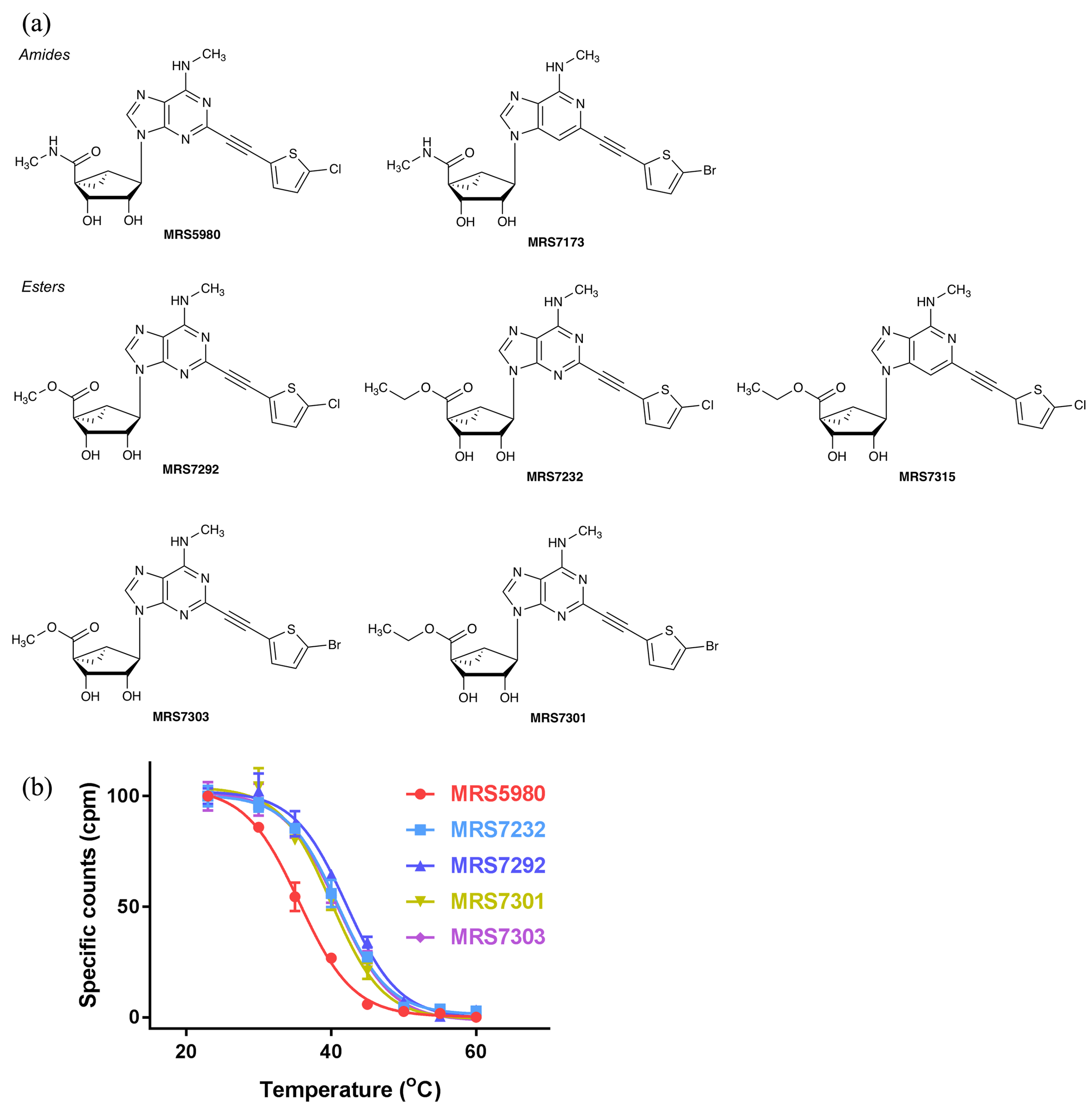

Supplement: S1 Fig — (a) Chemical structures of MRS compounds tested in this study for their effect on hDAT thermostability. (b) Tm of hDAT in complex with various MRS compounds. MRS7292 provides greatest enhancement in Tm. The error bars represent standard errors of mean calculated from triplicate measurements of a representative experiment (n = 2). (TIF) [file pone.0200085.s001.tif]

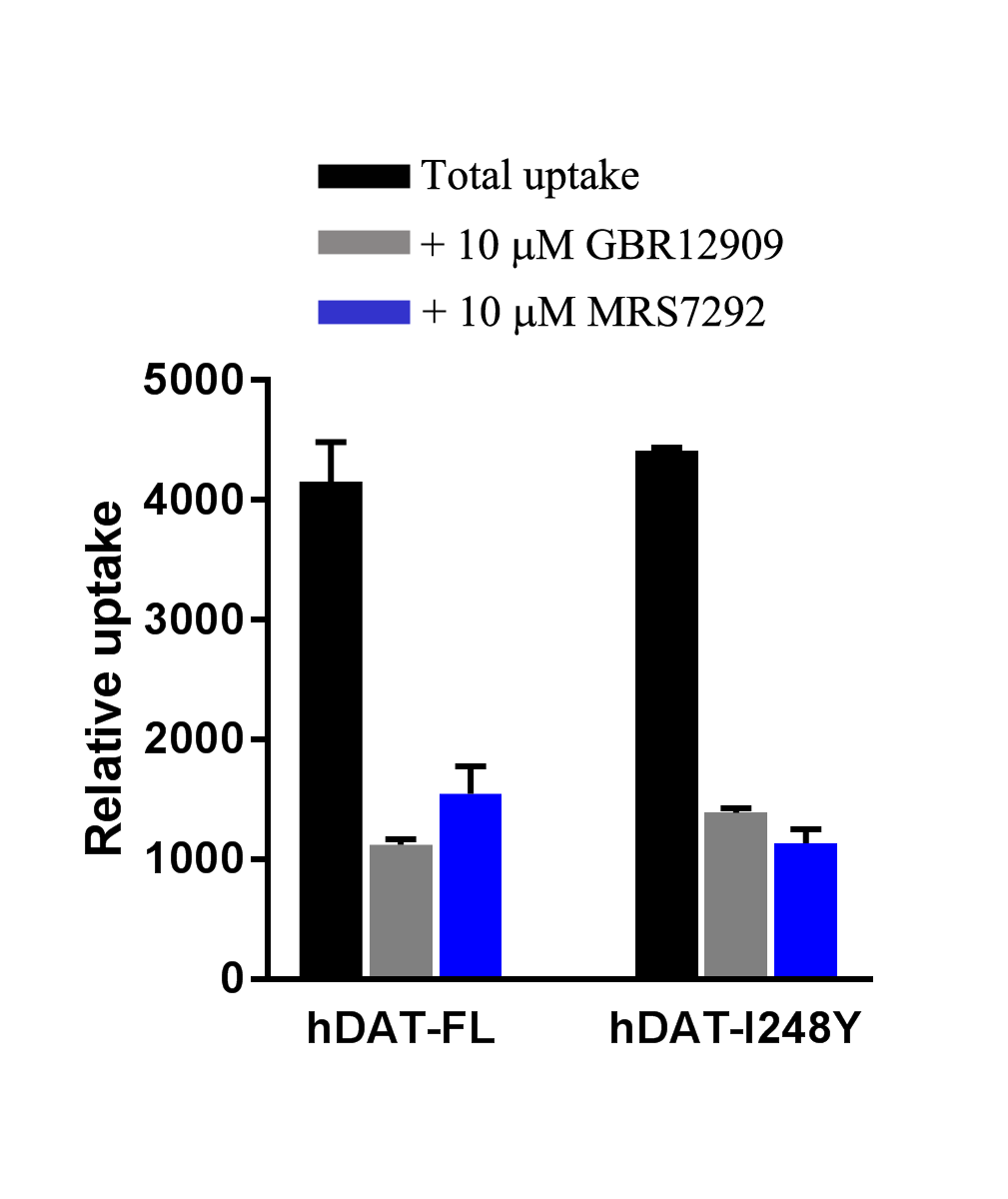

Supplement: S2 Fig — The thermostable hDAT-I248Y mutant is uptake active. The uptake counts displayed in the bar graph have been normalized with respect to expression levels of transporter. Also, the uptake was completely inhibited in the presence of MRS7292. The error bars represent standard errors of mean calculated from triplicate measurements of a representative experiment (n = 2). (TIF) [file pone.0200085.s002.tif]

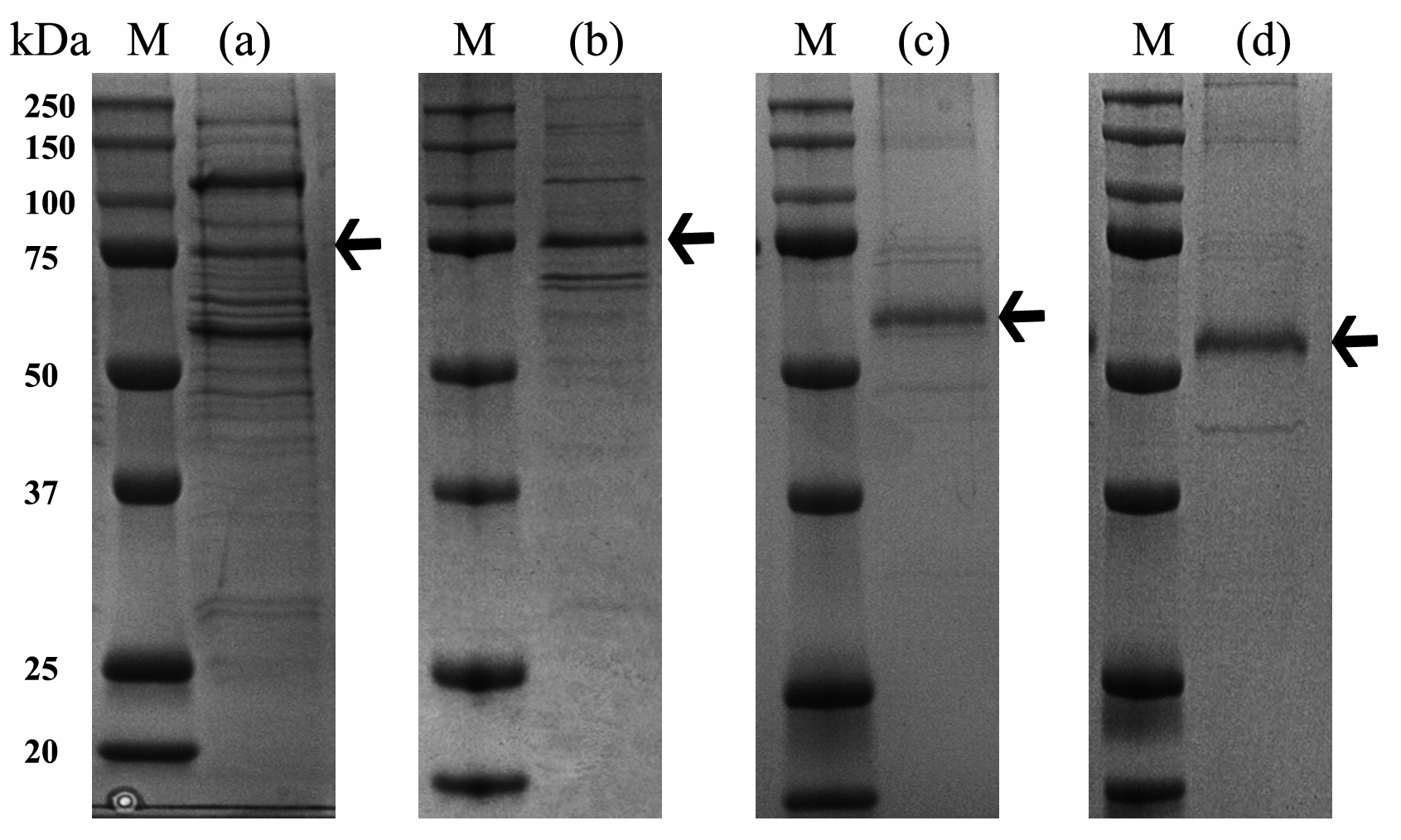

Supplement: S3 Fig — Purification (a) from whole cell solubilization using Talon affinity chromatography, (b) from solubilized membranes using Talon affinity chromatography, (c) using Strep-Tactin affinity chromatography, and (d) using NΔ56 expression construct and Strep-Tactin affinity chromatography. Position of hDAT in the lanes (a) & (b) were identified by in-gel GFP fluorescence. M denotes the protein molecular weight marker. (TIF) [file pone.0200085.s003.tif]

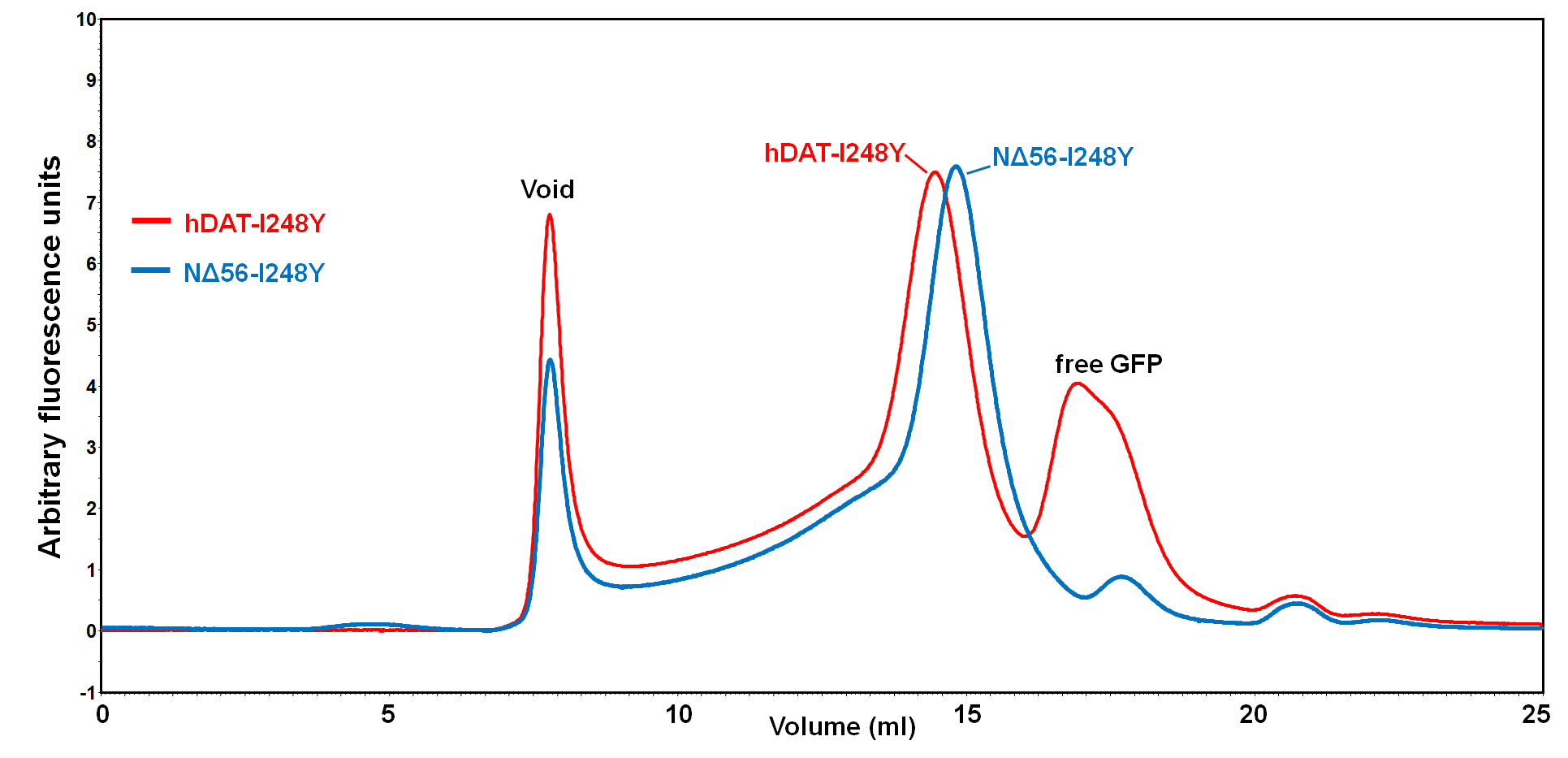

Supplement: S4 Fig — The N-terminal GFP fusion of hDAT-I248Y (red) and NΔ56-hDAT (blue) expression constructs are monitored by FSEC, using detergent solubilized cell lysate from HEK293S cells harvested 36 h post transfection. The NΔ56 expression construct shows smaller void and free GFP peaks than the full length construct. The free GFP is arising from non-specific proteolysis of N-termini. (TIF) [file pone.0200085.s004.tif]
